# Selective Laser Trabeculoplasty Versus Pattern Scanning Laser Trabeculoplasty for Reducing Intraocular Pressure in Primary Open-Angle Glaucoma

**DOI:** 10.7759/cureus.86050

**Published:** 2025-06-15

**Authors:** Jonathan Remon, Jessica M Diesing, Andrew J Boileau

**Affiliations:** 1 Medicine, Saba University School of Medicine, The Bottom, BES; 2 Neuroscience and Neurology, Saba University School of Medicine, The Bottom, BES

**Keywords:** intraocular pressure, ocular hypertension, pattern scanning laser trabeculoplasty, primary open angle glaucoma, selective laser trabeculoplasty

## Abstract

Selective laser trabeculoplasty (SLT) significantly lowers the intraocular pressure (IOP) in open-angle glaucoma patients compared to pattern scanning laser trabeculoplasty (PSLT). This review contains results from randomized control trials and a retrospective database analysis obtained through PubMed and Google Scholar. PubMed searches used the following words and terms: "selective laser trabeculoplasty" AND "pattern scanning trabeculoplasty.” A similar search on Google Scholar eventually uncovered one additional study comparing the two trabeculoplasty methods. All studies included patients diagnosed with primary open-angle glaucoma (POAG) or ocular hypertension.

SLT has not consistently shown superiority over PSLT in managing POAG. PSLT may result in slightly greater short-term reductions in IOP. Patients often report less discomfort with this method, suggesting it may be more tolerable. Both treatments can lead to meaningful reductions in IOP from baseline, which is generally considered a positive treatment outcome.

SLT and PSLT were shown to exert the same efficacy. In terms of treatment, both procedures are efficient and beneficial for lowering IOP. PSLT was favored to be more tolerable in one of the studies. Further research is required to observe potential side effects, long-term treatment, and mechanisms of action of both lasers.

## Introduction and background

Glaucoma is a common eye condition defined by its increased intraocular pressure (IOP) that can lead to blindness over time. It is the second leading cause of blindness in the United States, commonly found in older adults at the age of 40 and above [1]. This disease is known to cause acquired loss of retinal ganglion cells and axons within the optic nerve. Glaucoma can be further divided into different subtypes: primary or secondary, and the primary type can be further divided into open-angle or closed-angle depending on the obstruction inside the anterior chamber. A primary closed-angle glaucoma typically occurs when the flow of aqueous humor between the anterior and posterior chambers is blocked at the Schlemm’s canal by an enlarged iris [2]. Although not as prevalent, closed-angle glaucoma has a very swift progression to blindness if untreated. About 10% of all glaucoma cases in the United States are angle-closure glaucoma [3]. The more prevalent type, primary open-angle glaucoma (POAG), occurs with increased IOP due to blockage of the outflow tract between the posterior chamber and the iris. POAG is often asymptomatic, with some cases even having a normal IOP, with blindness developing over time [4]. In 2022, the prevalence of POAG was about 2%, with 250,000 persons between the ages of 40 and 49 years and 1.5 million persons above the age of 70 [5]. Most secondary types of glaucoma, whether they be open-angle or closed-angle, are usually due to eye trauma or an underlying medical condition such as congenital, pigmentary, or traumatic glaucoma [1]. For the purpose of this review, POAG will be the main subtype investigated.

Currently, multiple methods of treatment exist for POAG. The first and most used is in the form of IOP-lowering medications. Medications such as prostaglandin analogs, beta-blockers, alpha agonists, and carbonic anhydrase inhibitors all aid in lowering IOP through different mechanisms of action [6]. The other form of treatment greatly used is surgery, either incisional or laser. Incisional surgeries include trabeculectomy or the use of glaucoma drainage devices [7]. Trabeculectomy is done by creating a drainage space in the sclera to decrease IOP. A glaucoma drainage device is a procedure in which an implant in the eye serves as an alternate channel for aqueous humor that runs from the anterior chamber through a tube to an equatorial plate, which induces bleb formation [8]. Multiple types of laser trabeculoplasties exist, which include selective laser trabeculoplasty (SLT), pattern scanning laser trabeculoplasty (PSLT), argon laser trabeculoplasty, titanium sapphire laser trabeculoplasty, and micropulse laser trabeculoplasty. Each of these laser procedures induces changes in the trabecular meshwork through different methods of action. When it comes to treating POAG, a mixture of medication, incisional surgeries, and laser surgeries are used to effectively lower IOP and treat progressive blindness. In this review, two main types of treatment will be further explored: SLT versus PSLT.

SLT is a quick procedure that lasts for about 5 to 10 minutes per eye. The procedure begins with a patient sitting on a chair with their chin placed on a chin rest. The eyes are then numbed with the help of anesthetic eye drops. A small contact lens is placed on the eye, through which the laser will emit rapid pulses of light to induce changes in the trabecular meshwork [9]. There is current debate about how this laser treatment carries out its mechanism of action and is thought to be multifactorial [10].

PSLT is a relatively new method of lowering IOP using a computer-guided algorithm that allows for swift and precise treatment. PSLT uses a full 360-degree approach to treat the total circumference of the trabecular meshwork, administered in 16 steps with sequences of 32 segments [11]. Because of its computerized algorithm, PSLT aims to be less traumatic toward ocular tissues, suggesting a more tolerable procedure. In addition, it can be considered as an alternative intervention when open surgery cannot be performed [12]. For these reasons, PSLT may become a more favorable approach to lowering IOP.

Currently, SLT is a well-established treatment for POAG with proven efficacy in reducing IOP, whereas PSLT remains an emerging technology with limited clinical data. While the primary studies suggest comparable IOP-lowering effects with few adverse events, long-term outcomes remain unexplored. This review aims to compare the available data on SLT and PSLT to clarify their relative effectiveness, tolerability, and roles in POAG management. Furthermore, this review seeks to support clinicians in optimizing treatment decisions involving laser-based therapies for POAG.

## Review

Methods

**Figure 1:**
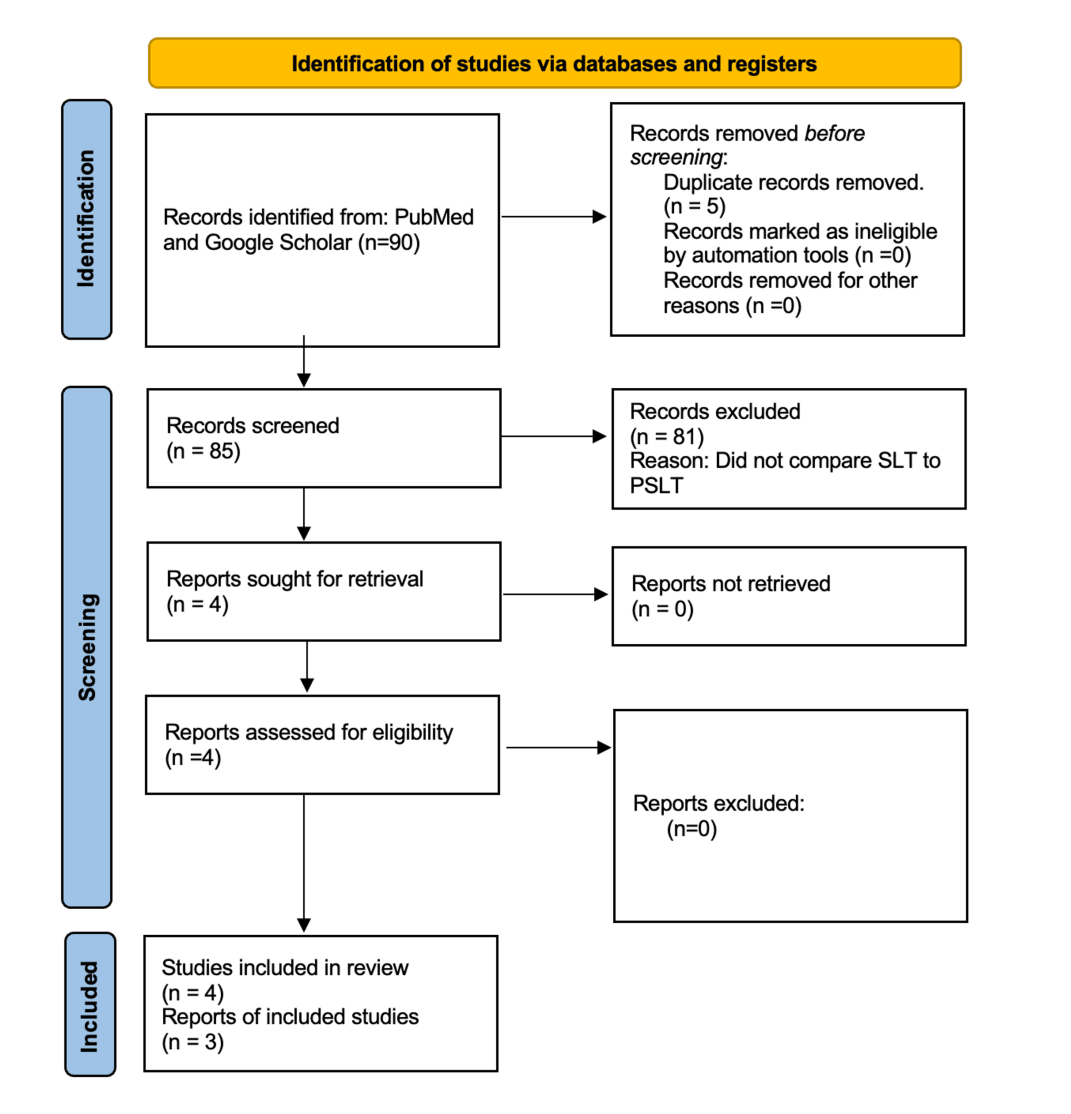
PRISMA-style flow diagram PRISMA: Preferred Reporting Items for Systematic Reviews and Meta-Analyses; SLT: selective laser trabeculoplasty; PSLT: pattern scanning laser trabeculoplasty

This literature review includes articles published in the past decade from the PubMed library database and from Google Scholar. Reviews and meta-analyses were excluded. Studies needed to have patients primarily diagnosed with open-angle glaucoma that were undergoing treatment with either SLT or PSLT and the trial must directly compare the two methods. An original Medical Subject Heading (MeSH) term was used to obtain the primary articles: ("Laser Trabeculoplasty" [MeSH]), but 0 results were found. An expanded search was created using the following terms: "Selective Laser Trabeculoplasty" AND "Pattern Scanning," which yielded five results. An advanced search in Google Scholar was formulated using the terms (pattern scanning selective laser trabeculoplasty open angle glaucoma) in the first row labeled “with all of the words.” Furthermore, in the second row labeled “with the exact phrase,” the string (“pattern scanning” AND “selective laser”) was inserted. In the row labeled “where my words occur,” the option “anywhere in the article” was toggled. This resulted in 90 articles. These articles were screened using the inclusion and exclusion criteria, and 81 articles were excluded for not comparing SLT to PSLT as reported in their abstracts. After a thorough review of the remaining literature, four articles were chosen for primary analysis, three of which were retrieved from PubMed. This information is summarized in Figure 1.

Results

Three randomized controlled trials and one retrospective data analysis were assessed to review and compare the effects of SLT and PSLT over the course of at least a year. Each paper measures IOP at baseline and through different time intervals after laser treatments. In addition, the percentage reduction from baseline was recorded after the operations. All papers included patients with a diagnosis of POAG. Since three out of the four papers were published in 2020, the results of the trials have been organized sequentially by month and year.

The study conducted by Mansouri and Shaarawy included 58 eyes from 29 patients [13]. All baseline parameters and clinical characteristics were similar in both PSLT and SLT groups. Patients were ruled into the study if they were 18 years or older and were diagnosed with primary or secondary open-angle glaucoma. They had to be either newly diagnosed, untreated for glaucoma, or had undergone a four-week washout period before the first study. Eyes were classified as glaucomatous if they had two consecutive abnormal perimetry test results and if they had abnormal-appearing optic discs, which were assessed with the use of slit lamp tonometry or spectral domain optical coherence tomography (OCT). Patients were excluded with previous laser trabeculoplasty, laser iridotomy, laser iridoplasty, or incisional glaucoma surgery. Randomization was completed with sequentially numbered sealed envelopes and distributed to PSLT or SLT by a study coordinator who did not participate in any further steps of the study. In addition, all eyes received a single session of 360-degree laser treatment [13].

PSLT was carried out with a Pascal Laser at a starting power of 500 mW and with the use of a custom-made PSLT gonioscopy lens to align the lasers on the trabecular meshwork. Titration was performed in most eyes that contained a visible degree of pigmentation in the inferior chamber angle. In those eyes where pigmentation was not visible, the PSLT procedure was done with 500 mW. SLT was performed with the Ellex Tango Laser. Starting power began with 0.6 mJ and was titrated to produce cavitation bubbles. Power was then decreased in increments of 0.1 mJ until there were only a few bubbles. The second eye was treated one week after the first eye. Laser treatment ranged from when the gonioscopy lens was applied to the eye until the end of treatment. Patients were given two drops of pilocarpine 2% before the laser treatment, and one drop of apraclonidine 1% was instilled after the laser treatment. Afterward, they were prescribed ketorolac tromethamine 0.5%, one drop four times daily for four days, and one drop of brimonidine twice daily for four days. Data recorded was analyzed using a two-tailed unpaired t-test, chi-squared test, Mann-Whitney U-test, and repeated measures analysis of variance. In addition, Kaplan-Meier survival analysis with the Mantel-Cox log-rank test was used to compare the results between the groups [13].

Patient comfort, assessed one hour after laser treatment with the visual analog scale (VAS), was 23.9 ± 20.5 cm (range: 0-82) in PSLT eyes and 50.4 ± 25.3 cm (range: 0-98) in SLT eyes (p<0.001), demonstrating better tolerability for PSLT. Measurements ranged from 0 (no discomfort) to 100 (very severe discomfort). Treatment was successful if IOP reduction was 20% or more in eyes that did not use ocular hypertensive medications (OHMs). In both groups, the effect of IOP reduction from baseline was statistically significant at all time points (p<0.01). The mean IOP at one month and three months for the PSLT group was 14.2 ± 2.5 mmHg and 13.9 ± 2.6 mmHg, respectively. The mean IOP for the STL group at one month and three months was 14.4 ± 4.1 and 13.7 ± 3.2, respectively. Although these values are very similar, the percentage of IOP reduction in the PSLT group was greater than the SLT group at one month (specific values were not shown) with a p-value of <0.01 and at three months with a 37% reduction in PSLT and 32% reduction in SLT (p<0.01) [13].

The study conducted by Rey et al. included 20 eyes from 14 patients in Bogota, Colombia, from June 2015 to June 2016 [14]. Inclusion criteria were defined as patients 40 years of age and older who were diagnosed with open-angle glaucoma or ocular hypertension with open-angle and had an IOP of 20 or more on two separate readings. Exclusion criteria were defined as any patient who had undergone any type of ocular procedure except iridotomy within the previous three months. The patients were randomized to either PSLT or SLT depending on which procedure would be covered by their insurance. Five minutes before laser treatment, patients were given a drop of brimonidine 0.2% in all treated eyes. In the PSLT group, all eyes were given a single session of 360-degree laser treatment. A Pascal laser was used with a starting power of 500 mW for 10 ms. Power was increased or decreased until light blanching on the trabecular meshwork was attained. After titration, power was maintained, but pulse duration was reduced to 5 ms to produce sub-visible lesions. SLT was executed using the OPTOSLT M laser. Starting power began at 0.6 mJ until cavitation bubbles were observed. It was then reduced in increments of 0.1 mJ until bubbles were minimal.

After laser treatment, patients were prescribed either topical diclofenac 0.1% or topical nepafenac 0.1%, one drop four times daily for four days. IOP measurements consisted of three readings recorded with Goldmann applanation tonometry (GAT). The averages of these three readings were then used in data analyses conducted with a two-tailed unpaired t-test, Mann-Whitney U-test, and repeated measures analysis of variance. An IOP reduction of 25% or more meant that treatment was successful [14].

Amongst patients, there were no statistical differences in age, gender, medication usage, or cup/disc ratio. IOP at baseline was 22.45 ± 2.4 for PSLT and 25.0 ± 2.2 for SLT. When comparing IOP in the SLT group versus the PSLT group, there was a significant decrease at one hour and 12 months in the PSLT group. The values for the PSLT group were 19.0 ± 5.0 mmHg at one hour and 13 ± 3.1 mmHg at 12 months, with a respective p-value of <0.01 at both time points. For the SLT group, the values were 25.6 ± 6.4 mmHg at one hour and 21.2 ± 4.3 mmHg at 12 months, with a respective p-value of <0.01 at both time intervals. When comparing the percentage reduction from baseline, the SLT group had a greater reduction from baseline at seven days with a value of 34.6 ± 14.7 mmHg as compared to the PSLT group, which had a value of 10.1 ± 31.1 mmHg with a respective p-value of <0.05 for both groups [14].

The retrospective database analysis completed by Elahi et al. contained data from a longitudinal monocentric database at the Glaucoma Research Center, Montchoisi Clinic, Swiss Visio, Lausanne, Switzerland [15]. Patients who had undergone laser trabeculoplasty before August 2017 and with a minimum follow-up of one year were also included in the Lausanne Laser Trabeculoplasty Registry. Exclusion criteria were defined by patients under the age of 40, those with a history of glaucoma laser or surgical intervention, and any diagnosis other than open-angle glaucoma or ocular hypertension. Each eye in PSLT was matched to an eye with SLT by baseline IOP, baseline number of OHMs, and glaucoma diagnosis. Baseline OCT-derived retinal nerve fiber layer, standard automated perimetry mean defect (MD), square root loss of variance, and best corrected visual acuity were recorded to determine the glaucoma stage. IOP using GAT and the number of medications were recorded at all visits.

The study included 280 eyes, of which 118 could not be matched 1:1. One hundred sixty-two eyes from 162 patients were divided equally amongst the SLT group and the PSLT group. PSLT was done using the Pascal laser with a custom-made gonioscope lens. SLT was performed with the Ellex Tango Laser and a traditional three-mirror contact gonioscope. The medications given during pretreatment and posttreatment as well as the laser procedures in this study were the same as the study conducted by Mansouri and Shaarawy [13]. There was no statistical significance in baseline characteristics between groups except the OHM baseline. Success of treatment was defined as an IOP reduction of 20% or more from baseline. The baseline IOP values were 1.4 ± 1.2 mmHg in SLT and 1.0 ± 1.0 mmHg in PSLT with a p-value of 0.05. Forty-five minutes postoperative, the PSLT group had a lower IOP than the SLT group. The values were 14.7 ± 3.3 mmHg in the PSLT group and 15.9 ± 3.4 mmHg in the SLT group, with a p-value of 0.04. In the first week postoperative, OHM values were 1.0 ± 1.2 mmHg in the SLT group and 0.6 ± 0.9 mmHg in the PSLT group with a p-value of 0.02 [15].

After a month, 102 out of the 162 eyes (53 SLT versus 49 PSLT) reached what was predetermined to be successful IOP reduction. However, 55 eyes (31 SLT versus 24 PSLT) failed to reach the success criteria due to additional procedures during the follow-up period. Among these 55 eyes, 48.4% (SLT) and 70.8% (PSLT) of them had repeat laser trabeculoplasty, while 51.6% (SLT) and 29.2% (PSLT) had intraocular surgery. These cases were then plotted onto a Kaplan-Meier survival curve to analyze the survivor function between SLT and PSLT. No statistical significances were noted between IOP or IOP reduction from baseline after procedures (p=0.09) or between survivor function of both lasers (p=0.74).

The randomized control trial performed by Wong et al. included 132 eyes from 132 patients with POAG after excluding 17 patients who did not meet the criteria [16]. The patients were divided into two groups: 67 SLT and 65 PSLT patients. Patients were randomized by even/odd designation generated from a random number table by a technician who was not involved in any other part of the study. Only patients, not ophthalmologists, were masked to treatment allocation. If both eyes of the patients were eligible, only one eye was randomized using an alternate right and left sequence, except for patients with visual field defects. The eyes with visual defects were randomized.

IOP was measured with GAT at one day, one week, and one, three, six, nine, and 12 months. In this study, PSLT was carried out with a Pascal laser with a drop of 0.1% of brimonidine 15 minutes prior to treatment. After instilling a drop of 0.5% proparacaine, the Latina 1X lens with coupling fluid was attached to the cornea, and the trabecular meshwork was visualized. Laser power and titration were repeated as in the previous studies. SLT was performed using the Selecta II Laser with a drop of 0.1% brimonidine instilled into the eye 15 minutes prior to treatment. After adding 0.5% proparacaine, the Latina 1X lens with coupling fluid was attached to the cornea, and the trabecular meshwork was visualized. Procedures for SLT laser and power were the same as the previous studies. Additional treatments included IOP-lowering medications such as prostaglandin analogs, beta-blockers, carbonic anhydrase inhibitors, or alpha-2 adrenergic agonists, which were prescribed if the IOP was still above 25 or more during the study follow-up and subsequent visits [16].

No significant differences were observed in baseline IOP or general characteristics such as age, spherical equivalent, visual acuity, visual field mean deviation, or retinal nerve fiber layer thickness. Day 1 postoperative had SLT with a lower IOP than the PSLT group. The values of IOP were 13.6 ± 4.1 mmHg for SLT and 15.4 ± 3.4 mmHg for PSLT-treated eyes with a p-value of 0.006. All patients had mild anterior chamber inflammation after PSLT/SLT, which subsided within one week to one month. One patient treated with SLT and one patient treated with PSLT had IOP increased more than 20% from baseline at day 1, contrasted with a 20% or more reduction, which was defined as successful treatment. One patient in the PSLT-treated group developed protracted anterior uveitis, which was resolved with the use of topical steroids for six months [16].

After performing multivariable models that were further limited to PSLT, the data showed a greater baseline IOP and a greater percentage of IOP reduction at day 1 to be associated with a greater percentage of IOP reduction at 12 months. Table 1 shows the factors associated with the percentage of IOP reduction at 12 months in patients who were treated with SLT from the study conducted by Wong et al. As shown in Table 1, there was statistical significance when observing the percentage of IOP reduction on day 1 when comparing it to the baseline that was measured with the GAT. All other factors assessed were not statistically significant. Table 2 shows the same comparisons but with the PSLT group. The percentage of IOP reduction at day 1, GAT IOP at baseline, and baseline visual field MD showed were statistically significant at 12 months for the PSLT group. All other parameters were shown to be statistically insignificant. The calculation for the percentage of IOP reduction at 12 months for both groups was completed by using the equation \[ \frac{\text{GAT IOP at baseline} - \text{GAT IOP at 12 months}}{\text{GAT IOP at baseline}} \times 100\% \] To calculate the percentage of IOP reduction on day 1, the equation is \[ \frac{\text{GAT IOP at baseline} - \text{GAT IOP at 1 day}}{\text{GAT IOP at baseline}} \times 100\% \]

**Table 1:** Factors associated with percentage of intraocular pressure reduction at 12 months in patients treated with SLT The adjusted R^2^ of the multivariable linear regression model is 0.41 *One item of IOP-lowering medication represents one class of IOP-lowering medication CI: confidence interval; SLT: selective laser trabeculoplasty; GAT: Goldmann applanation tonometry; IOP: intraocular pressure; MD: mean deviation

Factor	Value	95% CI	p-value
Age	0.02	-0.87 to 9.22	0.843
Percentage of IOP reduction at day 1 (every 10% decrease)	2.81	1.11 to 4.52	0.002
(GAT) IOP at baseline (every mmHg increase)	1.27	0.55 to 1.98	0.001
Baseline visual field MD (every dB increase)	-0.01	-0.69 to 0.68	0.985
*No. of medication at 12 months (every item increase)	4.17	-0.87 to 9.22	0.103

The values from these calculations are seen in Tables 1-2, which demonstrated a change in IOP reduction from baseline in both groups [16].

**Table 2:** Factors associated with percentage of intraocular pressure reduction at 12 months in patients treated with PSLT The adjusted R^2^ of the multivariable linear regression model is 0.50 *One item of IOP-lowering medication represents one class of IOP-lowering medication CI: confidence interval; PSLT: pattern scanning laser trabeculoplasty; GAT: Goldmann applanation tonometry; IOP: intraocular pressure; MD: mean deviation

Factor	Coefficient	95% CI	p-value
Age	-0.05	-0.21 to 0.26	0.71
Percentage of IOP reduction at day 1 (every 10% decrease)	2.74	0.47 to 5.01	0.019
GAT IOP at baseline (every mmHg increase)	2.28	1.36 to 3.20	<0.001
Baseline visual field MD (every dB increase)	-0.72	-1.42 to -0.01	0.048
*No. of medication at 12 months (every item increase)	4.26	-0.92 to 9.43	0.105

Discussion

PSLT does not have any major advantages over SLT. What has been observed through multiple studies, including the ones previously reviewed, is that laser trabeculoplasty exhibits stronger efficacy at higher baseline IOP. As seen in the study by Mansouri and Shaarawy, the patients had a low baseline IOP, which is a likely reason for the modest IOP-lowering effects after laser treatments [13]. As suggested by a large prospective trial on SLT by Pillunat et al., SLT may not work for baseline IOP levels below 14 mmHg [17]. However, the only predictive parameter for IOP reduction after SLT is to compare to baseline IOP. For this reason, Mansouri and Shaarawy's study had a few limitations [13]. The first limitation included patients with POAG with low IOP not categorized as normal tension glaucoma (NTG). In addition, some of these patients would technically have been classified as NTG with commonly used cutoffs such as IOP being equal to or less than 21. This cutoff can be observed in studies referenced by Lee et al. and Nitta et al., who used SLT in patients with NTG, concluding that SLT was still successful at lowering IOP [18,19]. Another limitation of the Mansouri and Shaarawy study was the follow-up period of one year [13]. To be able to assess the longevity of IOP-lowering effects in SLT versus PSLT, a longer period is required to properly observe any side effects from treatment, visual field parameters, and lifestyle changes. One beneficial aspect of this study was implementing a washout period in all patients taking IOP-lowering medications and remaining untreated for six months.

There seems to be one point of interest brought up by Mansouri and Shaarawy, which is the better tolerability patients had to PSLT over SLT [13]. The mechanisms of action of PSLT and SLT are not extensively known, but there are a few reasons why PSLT might be more tolerable. The first reason is regarding the total treatment area. The 400-micrometer-diameter circular spot in SLT covers an area 16 times larger than the 100-micrometer-diameter circular spot in PSLT. A larger area would mean more damage to the surrounding tissue and cells, leading to more inflammation and the release of inflammatory cytokines. The other reason the study mentioned is that PSLT has a shorter duration (5 ms), which reduces thermal diffusion of the treatment area and non-disruption of trabecular beams. However, this observation was done in comparison to argon laser trabeculoplasty in a study by Lee et al. conducted in cat eyes [20]. Conventional SLT pulse duration takes three nanoseconds, as seen in an article by Jha et al., which could mean it takes a shorter amount of time to complete treatment than with PSLT [21].

Although PSLT and SLT have demonstrated similar effects, the study by Rey et al. presents several inconsistencies, the first being a questionable interpretation of the results [14]. One-third of patients in the SLT group were lost to follow-up at 12 months. Secondly, a small sample of 10 eyes in the PSLT group and six eyes in SLT that were not randomized could have impacted the validity of the results. The inclusion criteria of patients also made it difficult to find suitable participants. As explained by Rey et al., hypertensive patients despite medical therapy are very rare in Colombia, as well as hypertensive POAG [14]. Another concerning point of this study is the statistical independence of the fellow eyes that were treated and included in the analysis. Only three out of six SLT subjects were treated in both eyes, which raises the question of whether treatment response in one eye can influence the fellow eye. The study calls attention to SLT having a crossover effect in the fellow eyes of treated patients with a statistically significant IOP-lowering effect in a retrospective review by Onakoya et al. and a study by Rhodes et al. [22,23]. It seems that the crossover effect is caused by systemic dissemination of pro-inflammatory chemical substances after SLT. These effects can then be produced in the contralateral eye. Although unclear as to how these lasers truly work, this study makes a good argument for the mechanism of action in SLT. SLT seems to stimulate the production of free oxygen radicals, causing peroxidation of the meshwork. In turn, this alters the intercellular junction, which modifies flow across the canal of Schlemm, leading to an increased level of cytokines that remodel the juxtacanalicular extracellular matrix. Since there is a lack of visible tissue scarring and damage in PSLT, this suggests a similar mechanism of action as SLT. A noticeable distinction of PSLT is the decreased area impacted on the trabecular meshwork with a larger amount of laser shots.

The study by Rey et al. was one of two studies that did not include a washout period in patients with IOP-lowering medications [14]. The study mentions that the multiple medications consumed by patients could undermine the results of laser trabeculoplasties. However, the study indicates that patients who had multiple glaucoma medications had an excellent response to both lasers. It is also important to mention that although there haven’t been many side effects noted from these lasers, IOP spikes after treatment can occur. On average, the total energy delivered per eye with PSLT is 3.5 J versus 0.1 J in SLT. This would indicate more frequent IOP spikes in PSLT; however, this was not the case. Only one patient in the SLT group had an increase of 5 mmHg after one hour of treatment, with zero patients having an IOP spike in the PSLT group. The cause of the IOP spike is unknown, but it could be due to trabeculitis from the laser. This could still happen in patients treated with PSLT, as seen in a study conducted by Turati et al. [24]. For future directions, this study needs an interpretation of the long-term effects of PSLT treatment after one year and with a larger sample size, just like the study managed by Mansouri and Shaarawy [13].

The second article, which did not include a washout period, was the retrospective database analysis by Elahi et al. [15]. Because of the nature of a retrospective analysis, there is a higher degree of incomplete data and loss to follow-ups than if it would have been a prospective study. Another important limitation of this study was the participant population. More than 95% of participants were of Caucasian origin, making any results difficult to apply to other ethnicities. Also, this study did not evaluate the quality of life in patients after treatment with laser trabeculoplasty.

The study by Elahi et al. [15] has two different opinions on the use of medications affecting SLT or PSLT. On one hand, the study references three articles that observed three types of medications as predictors of laser treatment success. For example, one study by Bruen et al. observed the effects of prostaglandins on SLT [25]. A second study conducted by Lee et al. analyzed the effect of carbonic anhydrase inhibitors on SLT [20]. The third study by Kurysheva et al. used general OHMs to observe an effect on SLT [26]. What was shown in these three studies is that the use of these medications did not correlate with the success of SLT. In turn, eyes that have not been exposed to medications respond better to laser treatments. This is also confirmed in a study by McIlraith et al., which compared SLT eyes after washout to SLT in treatment-naive eyes and found the latter to respond significantly better [27]. Laser trabeculoplasty is vital in the early stages of POAG and should therefore be used as primary treatment rather than IOP-lowering medications.

On the other hand, Elahi et al. mention that the lack of washout from IOP-lowering medications may be of minimal relevance to impacting the results [15]. Two studies referenced by Mao et al. and Martow et al. [28,29] mention that washout of eye drops, among other factors such as sex, diagnosis, and pigmentation of anterior chamber angle, is not associated with SLT efficacy. However, to get a true understanding of how SLT and PSLT can regulate IOP, these treatments should be used as primary methods of treating POAG or ocular hypertension and analyzed in a prospective cohort study without the use of medications. This can pose a harmful problem because some patients can still have an increase in IOP requiring further surgical treatment and the possible use of medications to control the IOP, as seen in one patient one week after laser treatment in the study by Mansouri and Shaarawy [13].

The issue that these studies have in common is assessing the long-term effects of laser treatments after one year. This problem is presented in the study by Wong et al., where quality of life was not measured due to a one-year follow-up being an insufficient amount of time [16]. Another limitation of this study was its patient population. In this case, only Chinese patients were assessed, making results hard to apply to other ethnicities. This study indicates that disparities in ethnicity, baseline IOP, follow-up duration, and subtypes of glaucoma among individuals referenced in the study by Mansouri and Shaarawy may contribute to the variation in IOP reduction following SLT [13]. This observation is also made in relation to the studies conducted by Turati et al. and Espinoza et al. [24,30], which are the few studies that have assessed the use of PSLT in patients with open-angle glaucoma.

The findings of baseline IOP and treatment response at day 1 are predictive of treatment response at 12 months in both PSLT and SLT [16]. This study suggests the state of patient prognosis can be defined with the use of baseline IOP. These parameters can be confirmed with the data collected by Wong et al. in Table 1 and Table 2 of the results. Table 1 shows the statistical significance of IOP reduction on day 1 from baseline IOP. Table 2 shows the same relation with the addition of baseline MD visual field to be affected significantly by PSLT. This data further supports the hypothesis made by the previous studies in which baseline IOP is the most important parameter to observe a change in IOP reduction with the use of laser trabeculoplasty.

Important side effects that occurred in the research by Wong et al. were two IOP spikes, one patient in SLT and one patient in PSLT, at day 1 post-laser treatment [16]. One patient developed anterior uveitis for six months but luckily experienced no clinical consequences from it. This study made sure to use a washout period of four weeks for any patients using IOP-lowering medications before recording their baseline parameters. Over 70% of patients remained medication-free at 12 months post-laser treatment. Further studies are required to properly assess the IOP-lowering effects of both laser treatments since PSLT is a rarely used method of laser trabeculoplasty. Pertinent data from the four primary studies can be appreciated in Table 3.

**Table 3:** Comparison of PSLT and SLT: summary of clinical studies PSLT: pattern scanning laser trabeculoplasty; SLT: selective laser trabeculoplasty; IOP: intraocular pressure; OH: ocular hypertension; POAG: primary open-angle glaucoma; VAS: visual analog scale; OHM: ocular hypotensive medication

First author	Date of publication	Study design	Level of evidence	Study population	Therapy or exposure	Outcome/results	Numerical findings
Rey et al. [14]	2020	Randomized control trial	1	Patients with hypertensive glaucoma	PSLT and SLT	IOP was lower in the PSLT group compared to the SLT group. PSLT and SLT were similar at 1, 3, 6 months but PSLT was more effective at 12 months.	PSLT: 19.0 ± 5.0 at 1 hour, 13 ± 3.1 at 12 months; SLT: 25.6 ± 6.4 at 1 hour, 21.2 ± 4.3 at 12 months (p<0.01). SLT had greater reduction at 7 days: 34.6 ± 14.7 versus PSLT 10.1 ± 31.1 (p<0.05).
Elahi et al. [15]	2020	Retrospective database analysis	3	Patients over 40 years old with ocular hypertension and open-angle glaucoma	PSLT and SLT	No significant difference between efficacy and safety in PSLT and SLT.	PSLT: IOP 14.7 ± 3.3; SLT: 15.9 ± 3.4 (p=0.04). OHM PSLT: 0.6 ± 0.9; SLT: 1.0 ± 1.2 (p=0.02). No difference in IOP/survivor function.
Mansouri and Shaarawy [13]	2016	Randomized control trial	1	29 patients with primary and secondary open-angle glaucoma	PSLT and SLT	IOP reduction in PSLT group was greater than SLT at 1 month and 3 months. Visual analog scale was better in PSLT group.	PSLT: IOP 14.2 ± 2.5 (1 month), 13.9 ± 2.6 (3 months); SLT: 14.4 ± 4.1 (1 month), 13.7 ± 3.2 (3 months). VAS: PSLT 23.9 ± 20.5; SLT 50.4 ± 25.3 (p<0.01).
Wong et al. [16]	2020	Randomized control trial	1	132 patients with POAG or ocular hypertension over a 12-month follow-up	PSLT and SLT	PSLT was not superior to SLT in safety or IOP-lowering efficacy.	SLT: IOP 13.6 ± 4.1; PSLT: 15.4 ± 3.4 (p=0.006). A higher baseline IOP and a greater percentage of IOP reduction at day 1 were associated with a greater percentage of IOP reduction at 12 months (p<0.001).

## Conclusions

Since the nature of SLT and PSLT is still unknown, further research would be beneficial in understanding how these lasers lower IOP in treated eyes and fellow eyes. Lifestyle modifications and side effects after one year of treatment should be critically considered to decide which form of laser treatment would be better long-term. On the other hand, these procedures are a great choice of treatment for lowering IOP in patients who have a high baseline IOP. Along with the use of medications and implants, SLT and PSLT should be implemented as primary forms of treatment. However, when comparing SLT and PSLT, few distinctions were observed, as both demonstrated similar efficacy.
